# Antibacterial activity of human defensins against *Staphylococcus aureus* and *Escherichia coli*

**DOI:** 10.7717/peerj.10455

**Published:** 2020-11-25

**Authors:** Albert Bolatchiev

**Affiliations:** Department of Clinical Pharmacology, Stavropol State Medical University, Stavropol, Russian Federation

**Keywords:** Antimicrobial peptides, Antibiotic resistance, HNP-1, hBD-1, hBD-3

## Abstract

**Background:**

The global problem of antibiotic resistance requires the search for and development of new methods of treatment. One of the promising strategies is the use of low doses of antimicrobial peptides, in particular, human defensins HNP-1, hBD-1, and hBD-3, in combination with antibacterial drugs already used in clinical practice. This approach may be used to increase the effectiveness of conventional antibiotics. However, this requires thorough study of the effectiveness of defensins in combination with antibiotics against a large number of bacterial strains with known phenotypes of antibiotic resistance. The aim of this work was to study the antibacterial effect of HNP-1, hBD-1 and hBD-3 in combination with rifampicin or amikacin against clinical isolates of *Staphylococcus aureus* (*n* = 27) and *Escherichia coli* (*n* = 24) collected from hospitalized patients.

**Methods:**

The standard checkerboard assay was used to determine minimum inhibitory concentrations (MICs) of antimicrobials. The combined microbicidal effects of two substances (defensin + conventional antibiotic) were assessed by the fractional inhibitory concentration index (FICI).

**Results:**

The highest anti-staphylococcal activity (including methicillin-resistant strains) among defensins was demonstrated by hBD-3 that had MIC of 1 (0.5–4) mg/L (hereinafter, MIC values are presented as median and interquartile range). The MIC of HNP-1 against *S. aureus* was 4 (2–8) mg/L; the MIC of hBD-1 was 8 (4–8) mg/L. Against *E. coli*, the most effective was also found to be hBD-3 that had MIC of 4 (4–8) mg/L; the MIC of HNP-1 was 12 (4–32) mg/L. The combinations of HNP-1 + rifampicin and hBD-3 + rifampicin demonstrated synergistic effects against *S*. *aureus*. Against *E. coli*, combinations of HNP-1 + amikacin and hBD-3 + amikacin also showed synergy of action.

## Introduction

Rapid and widespread increase in the resistance of microorganisms to antimicrobial drugs is known to present a serious problem and challenge to modern medicine (Roca et al., 2015; Li & Webster, 2018). The threat of increasing antibiotic resistance and methods to combat it are under active discussion at the level of the World Health Organization and the United Nations; in 2016, the “Global action plan to combat antimicrobial resistance” has been published. According to this document, the key objectives to solve this problem are the optimization of the use of antimicrobial drugs, as well as the development of new drugs (World Health Organization, 2015). Over the past 10 years, only several new antibacterial drugs have been introduced to the pharmaceutical market (Bassetti et al., 2013; Andrei, Droc & Stefan, 2019). An increase in antimicrobial resistance naturally leads to a decrease in the effectiveness of therapy and, as a result, an increase in the duration of treatment, an increase in mortality and financial expenses on treatment (Fair & Tor, 2014; Rolain et al., 2016). For example, 19,000 people die annually in the United States from infections caused by methicillin-resistant strains of *Staphylococcus aureus* (MRSA) (Fischbach & Walsh, 2009), while the annual financial expenses on treatment of this infection comprise $3 billion. According to the latest report from the Centers for Disease Control and Prevention (USA), the financial burden associated with increasing microbial resistance comprises about $55 Billion and 8 Million additional bed days (US CDC, 2019). It is estimated that by 2050 more than 10 million people will die annually from infections caused by resistant strains and by that time the global economy will lose about US $100 trillion due to this problem (O’Neill, 2016).

The formation of resistance takes place due to various causes and mechanisms. This is known to be a natural evolutionary process of adaptation of microorganisms to frequent contact with substances possessing antimicrobial properties (Martinez et al., 2009). The wide spread of antibiotic resistance is due to two factors—mutations and horizontal gene transfer (Martinez & Baquero, 2000).

The human body is in continuous contact with a large number of pathogenic and non-pathogenic microorganisms. In the process of evolution, defense mechanisms have formed that allow first to identify the pathogen and then, if necessary, to exercise adequate control of its further penetration and spread. These tasks are accomplished through the innate immune system which is capable (unlike the adaptive immunity system) of immediately recognizing and destroying infectious agents of various nature (Iwasaki & Medzhitov, 2015). The most important component of innate immunity is antimicrobial peptides (AMPs) with a length of 5 to ~100 amino acid residues. These peptides have a broad spectrum of antimicrobial activity against various infectious agents: bacteria, viruses, fungi and protozoa. Among the six kingdoms (bacteria, archaea, protists, fungi, plants, and animals), more than 3,000 AMPs have been identified by now (Wang, Li & Wang, 2016). Among AMPs, of great interest are human defensins: human neutrophil peptide-1 (HNP-1), human beta-defensin-1 (hBD-1), and human beta-defensin-3 (hBD-3), since they have a wide spectrum of antimicrobial activity (Pachón-Ibáñez et al., 2017). Since the outer surface of all bacteria has a negative charge (due to the presence of lipopolysaccharides and/or teichoic acids), positively charged and hydrophobic AMPs (in particular, defensins) nonspecifically “accumulate” on the surface of both gram-positive and gram-negative microorganisms. The antibacterial activity of defensins is believed to be related to membrane permeabilization of microorganisms (Kagan et al., 1990; Wimley & Hristova, 2020). However, some AMPs have been found to use alternative mechanisms of antimicrobial action (Matsuzaki et al., 1991; Mor & Nicolas, 1994; Oren & Shai, 1998; Chan, Prenner & Vogel, 2006). It has also been shown that HNP-1 can inhibit the synthesis of the bacterial cell wall by binding to precursor lipid II (De Leeuw et al., 2010).

Unfortunately, the introduction of native AMPs into clinical practice as a monotherapy for bacterial infections has a number of limitations: high synthesis cost, hemolytic activity, cytotoxicity for macroorganism, immunogenicity, and pharmacokinetic specifics (Moravej et al., 2018; Lei et al., 2019). To solve these problems, two approaches have been proposed: (i) modifying native AMPs (or designing new peptides with antimicrobial activity) (Lei et al., 2019), and (ii) using native AMPs at low doses in combination with conventional antibiotics (Zharkova et al., 2019).

In this work, we investigated the effectiveness of the combined use of human defensins HNP-1, hBD-1, hBD-3 and antibiotics (rifampicin and amikacin) against isolates of *S. aureus* and *Escherichia coli* collected from hospitalized patients.

## Materials and Methods

### Peptides and antibiotics

We used recombinant AMPs, human defensins HNP-1 (purity ≥ 92%), hBD-1 (purity ≥ 95%), hBD-3 (purity ≥ 98%) (Cloud-Clone, Katy, TX, USA), and conventional antibiotics, rifampicin (Belmedpreparaty, Minsk, Belarus) and amikacin (Sintez, Kurgan, Russia). The amino acid sequences and characteristics of the AMPs used in this work are provided in Table 1.

**Table 1:** Amino acid sequences and characteristics of defensins used.

Peptide	Amino acid sequence	Length (amino-acid residues)	Molecular weight	Charge	Hydrophobic residues (%)
HNP-1	ACYCRIPACIAGERRYGTCIYQGRLWAFCC	30	3.45 kDa	+3	53
hBD-1	DHYNCVSSGGQCLYSACPIFTKIQGTCYRGKAKCCK	36	3.94 kDa	+4	36
hBD-3	GIINTLQKYYCRVRGGRCAVLSCLPKEEQIGKCSTRGRKCCRRKK	45	5.17 kDa	+11	33

### Bacterial isolates

Twenty-seven *S. aureus* isolates and twenty-four *E. coli* isolates were identified and their antibiotic resistance phenotypes determined at the Department of Clinical Microbiology of the Center of Clinical Pharmacology and Pharmacotherapy (Stavropol, Russia) in accordance with the European Committee on Antimicrobial Susceptibility Testing (EUCAST) protocols using the standard disk diffusion test (The European Committee on Antimicrobial Susceptibility Testing, 2020). The resistance of *S. aureus* to cefoxitin (with zone diameter breakpoint <22 mm) was considered as a marker of methicillin resistance (The European Committee on Antimicrobial Susceptibility Testing, 2020).

Bacterial strains were collected from patients admitted to the intensive care department of the Stavropol State Regional Clinical Hospital (Russia) in 2020.

### Study of combined antimicrobial action of defensins and conventional antibiotics

To determine the minimum inhibitory concentrations of individual substances and to study the combined antimicrobial action of defensins and rifampicin/amikacin, we used the standard checkerboard assay (White et al., 1996; Orhan et al., 2005; Wiegand, Hilpert & Hancock, 2008; Pfaller et al., 2011) modified according to previous work (Bolatchiev et al., 2020).

Briefly, pure cultures of bacteria were cultured on solid nutrient media (mannitol salt agar; BioMedia, St. Petersburg, Russia) for 18–24 h at 37 °C. A fresh morning culture was used to prepare a saline suspension with the McFarland turbidity standard of 0.5, that is, the suspension had the concentration of the corresponding microorganism of approximately 1.5 × 10^8^ CFU/mL. A total of 0.1 mL of the resulting suspension was diluted in 9.9 mL of 2.1% Mueller–Hinton broth SIFIN Institut für Immunpräparate und Nährmedien, Germany; 21 g/L premade medium powder in accordance with the manufacturer’s instructions to produce an inoculum containing about 1.5 × 10^5^ CFU/mL. Then, the inoculum (100 μL per well) was added to the wells of a sterile 96-well plate with a U-shaped bottom (Medpolymer, St. Petersburg, Russia). After that, serial two-fold dilutions of two combinations of antimicrobial compounds under study (50 μL each) were introduced into the wells. For greater accuracy of the experiment, a quadruple control was carried out—three wells in each plate contained: (1) control-1, only 2.1% Mueller–Hinton broth (200 μL, without bacteria and without antimicrobial compounds); (2) control-2, inoculum only (200 μL, without antimicrobial compounds); (3) control-3, defensin at a maximum concentration without inoculum (200 μL); (4) control-4, rifampicin/amikacin at a maximum concentration without inoculum (200 μL). All antimicrobial compounds were dissolved in 2.1% Mueller–Hinton broth. In all experiments, the concentration range of antimicrobial substances was from 0 to 64 mg/L. Experiments with each of the microorganisms were carried out in at least three replicates. After the introduction of inoculum and antimicrobial substances, the plates were incubated in a thermostat at 37 °C. In 18–20 h, the presence or absence of growth was visually assessed. The minimum inhibitory concentration (MIC) was taken to be the lowest concentration of the test substance at which the growth of microorganisms was visually completely absent (Milly, Toledo & Ramakrishnan, 2005).

The combined microbicidal effect of two substances (A and B) was assessed by the fractional inhibitory concentration index (FICI) (Ruden et al., 2009): *FICI = (A/MIC A) + (B/MIC B)*, where A and B are such concentrations of antimicrobial agents in their mixture that inhibit the growth of bacteria; MIC A and MIC B are the minimum inhibitory concentrations of substances A and B, respectively, when they are applied separately. Depending on the FICI, there are three types of mutual influence of the two investigated antimicrobials on bacteria: (1) FICI *≤* 0.5—synergism of action; (2) 0.5 < FICI < 4—no interaction; (3) FICI > 4—antagonism (Sengupta et al., 2008).

The final MIC and FICI values were calculated as median values of three independent replicates (for each pair of antimicrobial compounds against each bacterial isolate).

## Results

All *S. aureus* isolates tested (*n* = 27) were susceptible to AMPs and rifampicin (Table 2). The MIC of HNP-1 for the studied staphylococci was 4 (2–8) mg/L (hereinafter, MIC and FICI values are presented as median and interquartile range in brackets). The MIC of hBD-1 was 8 (4–8) mg/L; that of hBD-3—1 (0.5–4) mg/L; that of rifampicin—0.008 (0.004–0.016) mg/L. As can be seen, the highest anti-staphylococcal activity among defensins was demonstrated by hBD-3.

**Table 2:** Minimum inhibitory concentration (MIC) of AMPs and rifampicin (RIF) against *S. aureus* isolates.

*S. aureus* isolates	Antimicrobial agent MIC (mg/L)				Resistance phenotype
	HNP-1	hBD-1	hBD-3	RIF	
SA-1	8	4	4	0.004	AMP, CIP
SA-2	4	8	0.5	0.004	CIP
SA-3	8	16	8	0.008	CIP
SA-4	4	2	0.5	0.004	AMP, CIP, ERY, AZM
SA-5	4	4	1	0.008	CIP, DOX
SA-6	0.5	1	1	0.004	CIP, DOX
SA-7	2	4	0.5	0.004	AMP, ERY, AZM
SA-8	4	8	1	0.008	AMP, AZM
SA-9*	0.5	4	0.5	0.016	FOX, AMP, CIP, LVX, DOX, ERY, AZM, GEN, AMN
SA-10*	0.5	8	1	0.016	FOX, GEN, AMN
SA-11*	4	2	4	0.016	FOX, AMP, GEN, AMN
SA-12*	2	16	8	0.016	FOX, AMP, CIP, LVX, DOX, ERY, AZM, GEN, AMN
SA-13*	2	4	4	0.008	FOX, AMP, CIP, LVX, DOX, ERY, AZM, GEN, AMN
SA-14*	8	4	1	0.008	FOX, AMP, CIP, LVX, DOX, ERY, AZM, GEN, AMN
SA-15*	4	8	0.5	0.008	FOX, AMP, CIP, LVX, DOX, ERY, AZM, GEN, AMN
SA-16*	4	16	0.5	0.004	FOX, AMP, CIP, LVX, DOX, ERY, AZM, GEN, AMN
SA-17*	16	2	1	0.008	FOX, AMP, CIP, LVX, DOX, ERY, AZM, GEN, AMN
SA-18*	4	2	0.5	0.004	FOX, AMP, CIP, LVX, DOX, ERY, AZM, GEN, AMN
SA-19*	1	2	0.5	0.004	FOX, AMP, CIP, LVX, DOX, ERY, AZM, GEN, AMN
SA-20*	4	16	0.5	0.032	FOX, AMP, CIP, LVX, DOX, ERY, AZM, GEN, AMN
SA-21*	4	8	1	0.016	FOX, AMP, CIP, LVX, DOX, ERY, AZM, GEN, AMN
SA-22*	16	4	4	0.016	FOX, AMP, CIP, LVX, DOX, ERY, AZM, GEN, AMN
SA-23*	4	16	0.5	0.004	FOX, AMP, CIP, LVX, DOX, ERY, AZM, GEN, AMN
SA-24*	16	8	0.5	0.032	FOX, AMP, CIP, LVX, DOX, ERY, AZM, GEN, AMN
SA-25*	4	8	1	0.008	FOX, AMP, CIP, LVX, DOX, ERY, AZM, GEN, AMN
SA-26*	16	8	4	0.016	FOX, AMP, CIP, LVX, DOX, ERY, AZM, GEN, AMN
SA-27*	4	16	2	0.032	FOX, AMP, CIP, LVX, DOX, ERY, AZM, GEN, AMN

**Notes:**

* MRSA.

FOX, cefoxitin; AMP, ampicillin; CIP, ciprofloxacin; LVX, levofloxacin; DOX, doxycycline; ERY, erythromycin; AZM, azithromycin; GEN, gentamicin; AMN, amikacin; RIF, rifampicin.

The results of MIC studies for *E. coli* isolates (*n* = 24) are presented in Table 3. In this case, the most effective AMP also was hBD-3 that had MIC of 4 (4–8) mg/L. The MIC of HNP-1 was 12 (4–32) mg/L. hBD-1 was found to be ineffective against 10 out of 24 *E. coli* isolates; its MIC against susceptible strains was 32 (14–32) mg/L. The MIC of amikacin was 3 (2–4) mg/L.

**Table 3:** Minimum inhibitory concentration (MIC) of AMPs and amikacin (AMN) against *E. coli* isolates.

*E. coli* isolates	Antimicrobial agent MIC (mg/L)				Resistance phenotype
	HNP-1	hBD-1	hBD-3	AMN	
EC-1	32	>64	8	4	AMP, AMC, CFX, IMP, CFS, GEN, LVX, CHL
EC-2	16	>64	4	2	AMP, AMC, CFX
EC-3	32	>64	4	4	AMP, AMC, CFX, IMP, LVX, CHL
EC-4	16	>64	4	4	AMP, CFX, CHL
EC-5	8	16	0.5	4	AMP, AMC, CFX, IMP, CHL
EC-6	4	32	1	1	AMP, CHL
EC-7	4	>64	4	4	AMP, CHL
EC-8	16	32	8	4	AMP, AMC, CFX, GEN, CHL
EC-9	8	32	16	4	AMP, CHL
EC-10	32	8	8	4	AMP, AMC, CFX, CHL
EC-11	32	16	4	2	AMP
EC-12	32	>64	2	1	AMP, AMC, CFX
EC-13	8	8	1	2	AMP, CHL
EC-14	4	32	2	4	AMP, AMC, CFX,
EC-15	2	>64	4	4	AMP, AMC, CFX, LVX, CHL
EC-16	4	32	8	2	AMP, CHL
EC-17	4	16	4	4	AMP, AMC, CFX, GEN, CHL
EC-18	8	8	8	2	AMP, CHL
EC-19	16	32	8	1	AMP, AMC, CFX, CHL
EC-20	32	32	4	1	AMP, CHL
EC-21	32	>64	8	2	AMP, AMC, CFX, IMP, LVX, CHL
EC-22	16	32	8	4	AMP
EC-23	8	>64	8	1	AMP, CFX, CHL
EC-24	4	>64	8	2	AMP, AMC, CFX, CHL

**Note:**

AMP, ampicillin; AMC, amoxicillin-clavulanic acid; CFX, cefotaxime; IMP, imipenem; CFS, cefoperazone-sulbactam; LVX, levofloxacin; GEN, gentamicin; AMN, amikacin; CHL, chloramphenicol.

We showed that against *S. aureus* (including MRSA), the combinations of HNP-1 + rifampicin and hBD-3 + rifampicin in most cases demonstrated synergistic effects—the FICI values for both combinations were 0.5 (0.375–0.5) (Table 4). The combination of HNP-1 + rifampicin did not show a synergistic effect against only 3 out of 27 *S. aureus* isolates (SA-4, SA-6, SA-19). When the combination of hBD-3 + rifampicin was used, the FICI value exceeded 0.5 for three isolates of *S. aureus* (SA-4, SA-9, SA-21), which indicates the absence of interaction between these substances against these strains. As to the combination of hBD-1 + rifampicin, we showed that only in 3 cases out of 27 there is a synergism of action (against isolates SA-5, SA-13, SA-14, see Table 4), while the median FICI value was 0.75 (0.75–1.25).

**Table 4:** Fractional inhibitory concentration indexes (FICI) of human defensins in combination with rifampicin (RIF) against *S. aureus* isolates.

*S. aureus* isolates	FICI		
	HNP-1 + RIF	hBD-1 + RIF	hBD-3 + RIF
SA-1	0.5	1.5	0.5
SA-2	0.5	1	0.5
SA-3	0.375	0.75	0.375
SA-4	0.75	0.75	0.875
SA-5	0.375	0.5	0.5
SA-6	0.625	1.5	0.5
SA-7	0.5	1.25	0.5
SA-8	0.5	0.75	0.375
SA-9*	0.375	0.75	0.625
SA-10*	0.5	0.625	0.5
SA-11*	0.375	1.25	0.3125
SA-12*	0.15625	0.75	0.375
SA-13*	0.5	0.5	0.5
SA-14*	0.5	0.5	0.375
SA-15*	0.3125	0.75	0.25
SA-16*	0.28125	0.625	0.5
SA-17*	0.375	0.75	0.375
SA-18*	0.5	1.25	0.5
SA-19*	0.625	1.0625	0.375
SA-20*	0.265625	1.125	0.375
SA-21*	0.375	1.5	0.75
SA-22*	0.25	1.25	0.5
SA-23*	0.5	1.25	0.5
SA-24*	0.5	0.625	0.375
SA-25*	0.5	1.125	0.375
SA-26*	0.3125	1.5	0.375
SA-27*	0.375	0.75	0.5

**Notes:**

* MRSA.

FICI ≤ 0.5—synergistic effect; 0.5 < FICI < 4—no interaction; FICI > 4—antagonism.

The study of the combined antimicrobial action of defensins with amikacin against *E. coli* isolates produced similar results. The combinations of HNP-1 + amikacin and hBD-3 + amikacin in most cases demonstrated synergistic action—the FICI values were 0.375 (0.375–0.5) and 0.5 (0.375–0.5), respectively (Table 5). The combined use of HNP-1 and amikacin did not show synergy in only 3 cases out of 24 (EC-6, EC-11, EC-13), and the combination of hBD-3 + amikacin—only in 2 cases out of 24 (EC-5, EC-13). The combined use of hBD-1 and amikacin against *E. coli* isolates did not show a synergistic effect: FICI = 1 (0.75–1.5). Moreover, in 10 cases out of 24, it was not possible to calculate the FICI value of this combination of substances, since the MIC of hBD-1 for these 10 isolates was >64 mg/L (Table 5).

**Table 5:** Fractional inhibitory concentration indexes (FICI) of human defensins in combination with amikacin (AMN) against *E. coli* isolates.

*E. coli* isolates	FICI		
	HNP-1 + AMN	hBD-1 + AMN	hBD-3 + AMN
EC-1	0.5	–	0.5
EC-2	0.375	–	0.375
EC-3	0.25	–	0.5
EC-4	0.375	–	0.5
EC-5	0.375	1	0.75
EC-6	0.75	1	0.375
EC-7	0.375	–	0.5
EC-8	0.5	1.5	0.5
EC-9	0.375	0.75	0.375
EC-10	0.5	0.375	0.5
EC-11	0.75	0.75	0.375
EC-12	0.5	–	0.5
EC-13	0.625	1	0.75
EC-14	0.375	0.75	0.5
EC-15	0.5	–	0.5
EC-16	0.5	1	0.5
EC-17	0.25	1.5	0.5
EC-18	0.5	2	0.5
EC-19	0.375	1.5	0.375
EC-20	0.5	1.5	0.5
EC-21	0.375	–	0.5
EC-22	0.25	1.5	0.375
EC-23	0.375	–	0.5
EC-24	0.3125	–	0.375

**Note:**

FICI ≤ 0.5—synergistic effect; 0.5 < FICI < 4—no interaction; FICI > 4—antagonism; in some cases of hBD-1 + AMN combination FICI has not been calculated—since MIC values of hBD-1 were > 64 mg/L.

## Discussion

The results obtained are of interest from several points of view. First, even though studies of the antimicrobial activity of HNP-1, hBD-1 and hBD-3 against *S. aureus* and *E. coli* have previously been conducted, a thorough analysis of their MIC and FICI values against a large number of clinical isolates with heterogeneous antibiotic resistance phenotypes has not been carried out. Second, the obtained data can be used to search for and develop new strategies for overcoming resistance to antimicrobial drugs used in clinical practice, since this work shows that, for instance, the MIC values of rifampicin and amikacin decrease by several times when they are used in combination with HNP-1 or hBD-3.

We showed that antibiotic resistance phenotype (including methicillin resistance) does not affect the sensitivity of the studied bacterial isolates to AMPs (Tables 2 and 3). It can be argued that the mechanism of antimicrobial action of the studied defensins is at least not associated with the targets against which conventional antibiotics are directed. The antimicrobial activity of positively charged defensins is realized when a certain threshold concentration of AMP molecules on the outer surface of the lipid membrane of a bacterial cell is reached, so that the tangential tension is compensated, which ultimately leads to the formation of pores of different structure (Sengupta et al., 2008). The differences in MIC values between different isolates of the same species may be due to differences in the structures of their cell walls.

In this work, we did not study the activity of defensins at concentrations above 64 mg/L; there is no need to carry out MIC analysis at such high concentrations, since the use of AMPs in practice has a number of limitations. Implementation of AMPs is complicated by the fact that they are rapidly degraded by peptidases (Steckbeck, Deslouches & Montelaro, 2014). This leads to their short duration of action and significantly affects the pharmacokinetics. Moreover, AMPs can have a cytotoxic effect on eukaryotic cells and cause hemolysis (Matsuzaki, 2009; Takahashi et al., 2010). Another problem with the large-scale use of AMPs is the high cost of their production (Pachón-Ibáñez et al., 2017). To solve these problems, several strategies have been proposed: modification of AMPs or creation of new peptides (with a short amino acid sequence) (Pachón-Ibáñez et al., 2017), stimulation of the production of endogenous AMPs by administration of low molecular weight compounds (Chen et al., 2020), implementation of low doses of AMPs to enhance the antimicrobial activity of conventional antibiotics (Zharkova et al., 2019).

The effectiveness of AMPs significantly varies in different studies and against different strains of the same species (Ganz et al., 1985; Turner et al., 1998; Yang et al., 1999; Sahly et al., 2003; Dürr, Sudheendra & Ramamoorthy, 2006; Wilmes et al., 2011; Xhindoli et al., 2016). It should be noted that there is still no standard that defines control points (criteria) of susceptibility or resistance of certain species of bacteria to a specific AMP. Therefore, it is necessary to conduct studies of the MIC and FICI values against a large number of clinical isolates.

Earlier studies by other groups have shown that some natural and novel synthetic AMPs can exhibit synergistic effects in combination with aminoglycosides or rifampicin (Pollini et al., 2017; Wu et al., 2017). The mechanism underlying the synergistic action of HNP-1/hBD-3 with rifampicin and amikacin is most likely to be related to the fact that AMPs facilitate the penetration of antibiotics into cells (Zharkova et al., 2019). Zharkova et al. (2019) have shown that often there is synergy between highly active AMPs targeting membranes (for example, protegrin 1, hBD-3) and antibiotics with intracellular targets (for example, gentamicin, rifampicin), which suggests an increase in bioavailability as the main model of such interaction.

The implementation of low doses of AMPs can reduce the MICs of some antibiotics, which has been shown in numerous studies. For instance, the combination of hBD-3 and methicillin demonstrates a synergistic effect against clinical strains of MRSA with FICI values in the range of 0.09-0.45 (Midorikawa et al., 2003). This is very interesting because the use of methicillin alone is not effective against MRSA (The European Committee on Antimicrobial Susceptibility Testing, 2020), thus, hBD-3 can help overcome the resistance of MRSA to beta-lactam antibiotics. Similar results can be obtained for other combinations of AMPs with antibiotics to which the bacteria have acquired resistance. This would require studies with reference strains, followed by verification with respect to a large number of appropriate clinical isolates.

It has previously been shown that hBD-3 can effectively combat MRSA biofilms by suppressing bacterial growth, regulation of inflammation and immune responses in vivo (Zhu et al., 2017). In general, the effects of AMPs in vivo are very diverse: from wound healing (Bolatchiev et al., 2020) to the ability to neutralize the lethal toxin of the anthrax pathogen (Kim et al., 2005). Defensins can be considered as an effective link between innate and adaptive immune responses (Colavita et al., 2015), since AMPs directly stimulate the migration of immune cells, promote the release of pro-inflammatory cytokines, and activate antigen-presenting cells through the Th1 immune response (Suarez-Carmona et al., 2015). Thus, it is obvious that synergistic effects should be assessed in in vivo experimental models.

Thinking about further strategies for using these defensins to solve the problem of antibiotic resistance, we suggest that one of the approaches to future clinical applications of AMPs may be the search for ways to produce endogenous AMPs (for instance, by introducing low molecular weight compounds or viral vectors encoding peptide sequences) in combination with conventional antibiotics. On the one hand, this strategy can help to increase the effectiveness of antibacterial drugs, and on the other hand, the stimulation of endogenous AMPs is a much cheaper way of application of AMPs.

## Conclusions

Thus, in this work, we investigated the antimicrobial activity of human defensins HNP-1, hBD-1, and hBD-3 against clinical isolates of *S. aureus* (*n* = 27) and *E. coli* (*n* = 24). Among the studied defensins, HNP-1 and hBD-3 were the most effective. Moreover, these antimicrobial peptides showed a synergistic effect against most of the studied isolates when applied together with rifampicin and amikacin.

## Supplemental Information

10.7717/peerj.10455/supp-1 Supplemental Information 1 The raw data of MICs and FICIs calculations for all the studied bacterial strains.

## Additional Information and Declarations

### Data Availability

The following information was supplied regarding data availability: The raw measurements are available as a Supplemental File.
